# The Impact of Chinese College Students' Perceived Stress on Anxiety During the COVID-19 Epidemic: The Mediating Role of Irrational Beliefs

**DOI:** 10.3389/fpsyt.2021.731874

**Published:** 2021-09-08

**Authors:** Zhang Chi, Liu Qian, Liu Haihua, Lin Nuoxun

**Affiliations:** ^1^Center for Students' Psychological Quality Education, Beijing Jiaotong University, Beijing, China; ^2^Student Funding Center, Peking University, Beijing, China; ^3^Faculty of Social Sciences, Lingnan University, Tuen Mun, China

**Keywords:** perceived stress, anxiety, irrational beliefs, COVID-19, college students

## Abstract

**Objective:** To explore the underlying mechanism of the impact of perceived stress on anxiety of the Chinese college students during the COVID-19 epidemic.

**Methods:** The Perceived Stress Scale, Irrational Belief Scale, and General Anxiety Scale were adopted in the current study. College students were randomly selected for online questionnaire survey. There were 1,598 valid questionnaires, and the proportion of women was 47.81%.

**Results:** The perceived stress and anxiety, as well as the three dimensions of irrational beliefs (catastrophizing, low frustration tolerance, and depreciation) were significantly positively correlated; demandingness was not significantly correlated with anxiety. Further analysis found that the perceived stress had a significant positive predictive effect on the anxiety of college students. Catastrophizing, low frustration tolerance, and depreciation played part of the mediating role, and there was no significant difference in the strength of these mediating roles.

**Conclusion:** The perceived stress of the COVID-19 epidemic had a positive effect on the anxiety of Chinese college students, this was partly mediated by irrational beliefs.

## Introduction

On January 30, 2020, the World Health Organization (WHO) declared the outbreak of COVID-19 to be a Public Health Emergency of International Concern (PHEIC) (1). COVID-19 spreads quickly around the world with a comparatively high infection rate. It threats the world by the lack of effective vaccines or specific remedies and its high fatality rate (2). Studies have shown that the physical damage caused by such public health emergencies can be recovered in a short period of time, but the psychological damage will exist for a long period (3). After the SARS epidemic in China in 2003, a large number of patients with mental illnesses such as acute stress disorder and post-traumatic stress disorder appeared (4). Therefore, it is very necessary to provide the public with psychological support as soon as possible in response to the COVID-19.

In order to stop the spread of the COVID-19 epidemic into campuses, the Ministry of Education of the People's Republic of China requested universities to postpone the start of the 2020 spring semester (5). For college students, the epidemic changed their previous learning patterns and social styles. Worries about academic performance lead to dual stresses upon students' mental health (6). A volume of literature found that anxiety mostly followed a stressful event (7–10). Previous research found that compared with objective stress, subjective stress could better predict the mental health of college students (11). Therefore, the first question that this research intended to explore was whether college students' perceived stress during the epidemic would cause anxiety.

Perceived stress is a person's perception of threatening stimulus or unfavorable factor (12). It would cause confusion, the sense of being threatened, and challenged on the individual, in turn, the person might be in a state of tension or out of control (13). The development and magnitude of the perceived stress depends on a large extent on the individual's cognitive evaluation of environmental stimuli. Different people have different irritability to the same environmental stimulus based on different extent of their irrational beliefs (14, 15).

Irrational Beliefs (IBS) is the core concept of Rational Emotional Behavior Therapy (REBT) proposed by Albert Ellis, which has been widely used in psychological counseling and clinical treatment (16). Irrational beliefs refer to the rigid beliefs that things “should be” or “must be” based on a distorted understanding of objective things, or on the basis of illogical reasoning. In short, it is the unrealistic appraisal and evaluation of adverse events. Irrational beliefs are the absolute requirements and distorted views of oneself, others, and the surrounding environment. Irrational beliefs are usually divided into four categories: demandingness, catastrophizing, low frustration tolerance, and depreciation (17).

Previous research on the relationship between irrational beliefs and anxiety discovered that negative emotions such as depression and anxiety are closely related to irrational beliefs (18, 19). The fewer the individual's irrational beliefs, the lower the degree of anxiety. Irrational beliefs have impact on individual's interpretation styles (20). Individuals holding irrational beliefs are more likely to have the rigid demandingness belief, catastrophizing belief, low frustration tolerance, and depreciation belief than individuals holding rational beliefs, thus, have higher levels of anxiety (21, 22). Therefore, the second research question of the current study was that, whether irrational beliefs have a mediating effect on the relationship between the perceived stress and anxiety of college students during the COVID-19 epidemic.

As aforementioned, if the perceived stress affected the irrational beliefs of college students during the COVID-19 epidemic, meanwhile, the irrational beliefs affected their anxiety level, we thus hypothesized that irrational beliefs play the mediating role between the perceived stress and the anxiety of college students during the epidemic. Which is that perceived stress affects the anxiety of college students through the mediation role of their irrational beliefs. Previous studies have found that different types of irrational beliefs have different characteristics, which lead to different degrees of impact on mental health (23). The third research question that the current study wanted to explore was whether there were differences in the mediating effects of different types of irrational beliefs between the perceived stress and the anxiety of college students.

In sum, the current research hypothesized a parallel multiple mediation model to analyze the parallel mediation effects of different types of irrational beliefs. By so doing, to investigate the underlying mechanisms of perceived stress on anxiety of Chinese college students during the COVID-19 epidemic. This might evolve our understanding about the destructive factors within college students' responses to the stress caused by the epidemic. The evaluation and measurement of irrational beliefs will have instructive significance for clinical psychological counseling.

## Method

### Procedure and Participant

The current study was conducted in five universities in Beijing City on April 19, 2020. We distributed 1,800 pieces of questionnaires online, among which 202 invalid questionnaires were removed. In total, 1,598 (valid ratio = 89.8%) college students (47.81% were women) ranged from freshman to junior completed this online survey. A consent form was stated at the beginning of each questionnaire. The average age was 19.8 ± 1.3 year-old, median age 20 years old, range 16–25 year.

### Measures

#### The Perceived Stress Scale

The 10-item perceived stress scale was applied to measure the degree of stress experienced by an individual in the past 1 month (12). A sample item was “In the past month, you have been upset by unexpected events.” Answers were provided on a 5-point frequency scale, ranging from 0 (never) to 4 (always). The total score of the scale represented the degree of perceived stress: the higher the score, the stronger the perceived stress. The Cronbach's alpha was 0.79.

#### The Irrational Beliefs Scale

The 15-item Irrational beliefs scale was adopted from Wang Yu's irrational belief scale about college students based on the existing irrational belief scale (24). A sample item was “Any mistake will lead to great disaster.” Answers were provided on a 5-point frequency scale, ranging from 1 (strongly disagree) to 5 (strongly agree). The Cronbach's alpha was 0.60 of the dimension of demandingness, 0.61 of the dimension of catastrophizing, 0.70 of the dimension of low frustration tolerance, and 0.78 of the dimension of depreciation.

#### The Generalized Anxiety Disorder Scale (GAD-7)

The 7-item Generalized anxiety disorder scale was applied to measure the frequency of generalized anxiety disorder symptoms of participants in the last 2 weeks (25). Answers were provided on a 4-point frequency scale, ranging from 0 (not at all) to 3 (nearly every day). The total score of the scale ranged from 0 to 21, the higher the score, the more severe generalized anxiety disorder symptoms. Scores equal to or >10 indicate the diagnose of GAD. Scores ranged from 6 to 9, 10 to 14, 15 to 21 might represent the mild, moderate, and severe levels of anxiety on the GAD-7 (25). The Cronbach's alpha was 0.91.

### Data Analysis

SPSS 21.0 was applied to conduct the data analysis. Model 4 of the PROCESS (26) was applied to conduct the multiple parallel mediation analysis in testing the significance of the overall mediation and individual indirect effects of the catastrophizing, low frustration tolerance, and depreciation. The bootstrapping technique was applied to examine the significance of the hypothesized indirect effects. Bias-corrected 95% confidence intervals (BC 95% CIs) were computed based on 5,000 bootstrap resamples.

## Results

Harman's single-factor test was applied to detect the possible common method variance (CMV) on the current self-reported data (27). The results showed that there were 11 eigenvalues >1. The first common factor estimated the common method variance to be 21.74%, which is less than the threshold of 40%. Therefore, suggested CMV did not exist (28).

### Descriptive Statistics

Means, standard deviations, and correlations among the study variables were presented in Table 1. The correlation between demandingness and anxiety was not significant, thus demandingness was removed from the mediation analysis. The correlation between gender and anxiety was not significant, thus gender was removed from the control variables.

**Table 1:** Means, SD, and correlations between the study variables.

**Variable**	**M**	***SD***	**1**	**2**	**3**	**4**	**5**	**6**	**7**
1 Gender	–	–	–						
2 Perceived stress	26.743	5.629	0.012	–					
3 Anxiety	11.393	3.954	−0.018	0.654^***^	–				
4 Catastrophizing	8.621	2.067	−0.028	0.246^***^	0.274^***^	–			
5 Low frustration tolerance	12.635	2.692	0.025	0.309^***^	0.324^***^	0.414^***^	–		
6 Depreciation	11.399	3.215	−0.005	0.424^***^	0.401^***^	0.468^***^	0.543^***^	–	
7 Demandingness	12.401	1.718	0.112^***^	−0.087^**^	−0.015	0.213^***^	0.252^***^	−0.002	–

** *p < 0.01 and*

*** *p < 0.001*.

### Test of the Mediation Effect

**Figure 1:**
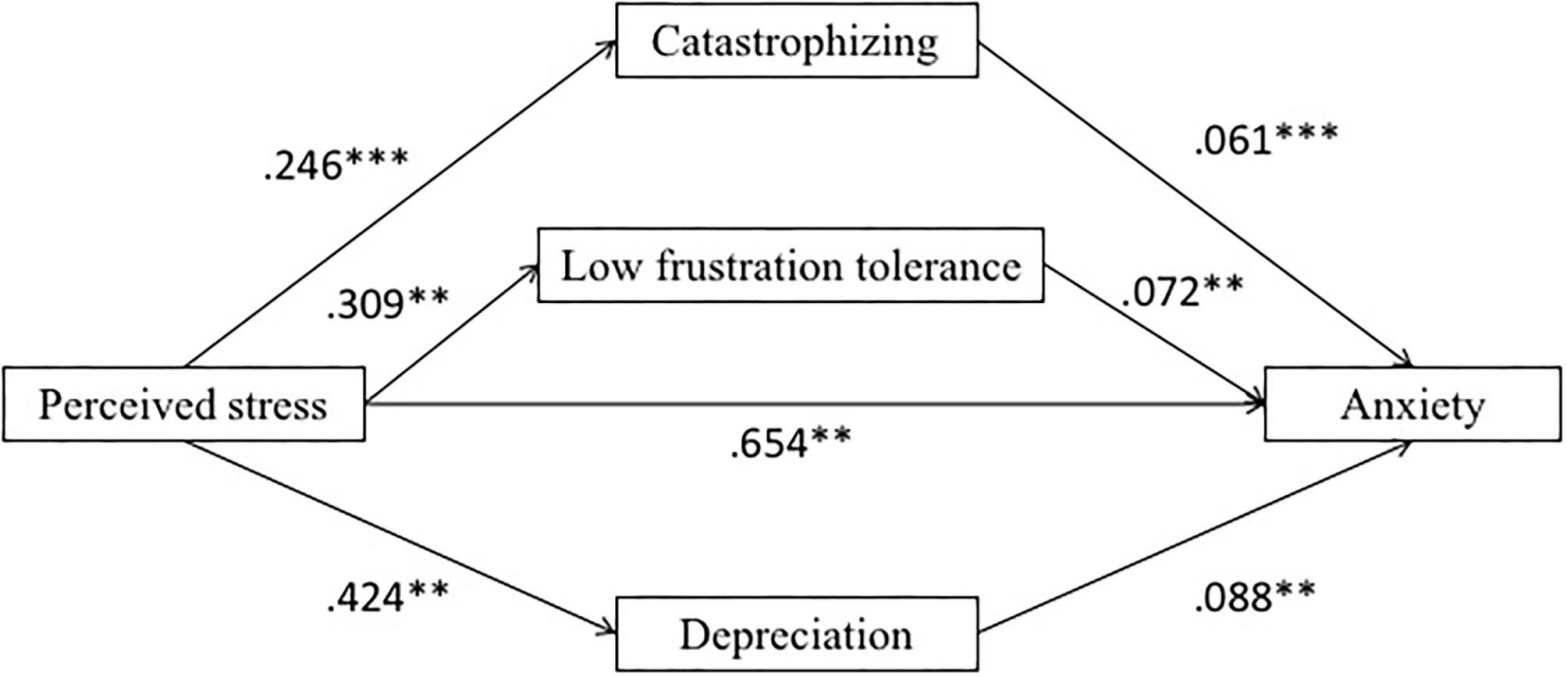
Multiple parallel mediation model of the current study. **p < 0.01 and ***p < 0.001.

Based on the results of correlation analysis, the current study intended to explore the mediating role of irrational beliefs (catastrophizing, low frustration tolerance, and depreciation) during the epidemic period between the perceived stress and anxiety of college students (see Figure 1 for the multiple parallel mediation model). Anxiety was the dependent variable, perceived stress was the independent variable, and irrational beliefs (catastrophizing, low frustration tolerance, and depreciation) were the mediating variables.

Results showed that perceived stress was positively related to anxiety (β = 0.65, *p* < 0.001). Perceived stress was positively related to catastrophizing (β = 0.25, *p* < 0.001), low frustration tolerance (β = 0.31, *p* < 0.001), and depreciation (β = 0.42, *p* < 0.001), respectively.

After integrating irrational beliefs as the intermediary variable, the positive predictive effect of perceived stress on anxiety was significant (β = 0.58, *p* < 0.001). Catastrophizing was positively related to anxiety (β = 0.06, *p* < 0.01), low frustration tolerance was positively related to anxiety (β = 0.07, *p* < 0.01), and depreciation was positively related to anxiety (β = 0.09, *p* < 0.001).

The total effect of perceived stress on anxiety was 0.459 (*p* < 0.001, LLCI = 0.4334, ULCI = 0.4855). The mediating effects of the three types of irrational beliefs were all significant, respectively. The mediating effect of catastrophizing estimated 2.40% (0.011/0.459) of the total effect; the mediating effect of low frustration tolerance estimated 3.49% (0.016/0.459) of the total effect; the mediating effect of the depreciation estimated 5.66% (0.026/0.459) of the total effect. In total, the mediating effect estimated 11.33% (0.052/0.459) of the total effect (see Table 2).

**Table 2:** Mediation analysis of irrational beliefs between perceived stress and anxiety.

	**Estimate**	**Boot SE**	**95% CI**	
Total indirect effect	0.052	0.008	0.037	0.068
Indirect effect 1	0.011	0.004	0.003	0.018
Indirect effect 2	0.016	0.005	0.006	0.026
Indirect effect 3	0.026	0.009	0.010	0.044
Comparison 1	−0.005	0.006	−0.018	0.008
Comparison 2	−0.016	0.010	−0.036	0.003
Comparison 3	−0.011	0.011	−0.034	0.011

*Indirect effect 1, Perceived stress → Catastrophizing → Anxiety; Indirect effect 2, Perceived stress → Low frustration tolerance → Anxiety; Indirect effect 3, Perceived stress → Depreciation → Anxiety; Comparison 1, indirect effect 1—indirect effect 2; Comparison 2, indirect effect 1—indirect effect 3; Comparison 3, indirect effect 2—indirect effect 3*.

## Discussion

The current study examined the relationships between the perceived stress, irrational beliefs, and anxiety among Chinese college students during the COVID-19 epidemic. Results revealed three mediation paths of perceived stress on anxiety through the mediation role of catastrophizing, low frustration tolerance, and depreciation, respectively. This research contributed to educators in reducing the anxiety of college students from the perspective of modifying their irrational beliefs.

### Theoretical Implication, Limitation, and Future Research

Individuals' irrational beliefs are closely related to their mental health (15). Previous literature such as Weng et al. (29) found that irrational beliefs could predict the degree of depression in patients with depression. Among college students, stress, and irrational beliefs were significantly related to alcohol issues (30). Irrational beliefs were proved to have a direct restrictive effect on the acquisition of social support and mental health (21). Based on previous studies, the current study advanced our knowledge of how anxiety, which is more common than depression during the epidemic (31), was affected by irrational beliefs.

Previous research has proved that stress had a significant predictive effect on the level of teacher's anxiety, through which irrational beliefs played the mediating role (32). The current study discovered this similar phenomenon among college students. In particular, under the urgent social context of the COVID-19 pandemic, extending our knowledge of how cognitive interpretation of the person-environment interaction influence individuals' mental health. In another study, the “low frustration tolerance” factor of irrational beliefs had the most predictive effect on high level of stress responses (24). Future research could pay more attention in exploring the mechanisms of how these three dimensions affect college students. In addition, the dimension of demandingness was not significantly correlated with students' anxiety, which was inconsistent with the notion of irrational beliefs (33). Future study would be necessary to verify this correlation. Moreover, the current research measured the general irrational beliefs of the college students' instead of their specific irrational beliefs which is closely related to the COVID-19 pandemic. Future research should pay attention to adapt the measure to detect the specific cognitive interpretations on the targeted adverse event.

The current research adopted the survey method in data collection, which belongs to the category of quantitative research in the research paradigm and lacks qualitative analysis. In-depth interviews combined with individual cases and follow-up research will be the direction of future research.

Finally, our results draw on the cross-sectional data in investing the mediation paths, even though we recruited a large sample size, this design is not efficient enough to support the causal relationships (34). Future study should consider the longitudinal survey or intervention design in replicating the current results.

### Practical Implication

It is of significant importance to cognitively guide college students to help them detect and change unreasonable cognitive styles and beliefs, and respond calmly and rationally to the psychological impact of the major public health emergency, such as the COVID-19, as well as other future adverse events in daily life. Maintaining a positive and optimistic attitude instead of blindly catastrophizing the results of a stressful event, might reduce their risk of psychological symptoms (18, 20). Improving tolerance about setbacks by rationally understand the causes of stressful events, so as to be able to develop adaptive and effective coping strategies. By so doing to treat setbacks as an important opportunity to practice their mental endurance, and improve their mentality adaptability. Taking a comprehensive view of stressful events, such as to maintain a calm mind, neither underestimate the harm of stressful events, relax vigilance; nor exaggerate the risks of stressful events, and create artificial tension and panic. The current research reveals the cognitive processes of why college students suffer from anxiety during such a public health emergency, pointing out the foci where practitioners could work with college students to help them suffer less from anxiety.

## Conclusion

The current study investigated a parallel mediation model of the relationships among perceived stress, anxiety, and irrational beliefs (demandingness, catastrophizing, low frustration tolerance, and depreciation). Results revealed the mechanism of Chinese college students when encountering the outbreak of COVID-19 pandemic. The current research explored the destructive factors of college students' psychological response to the stress caused by the epidemic. On the one hand, it added to our knowledge of the underlying cognitive interpretations between perceived stress and anxiety. On the other hand, it has significant practice value on the interventions about how to maintain individuals' mental health when facing public health emergency.

## Data Availability Statement

The original contributions presented in the study are included in the article/supplementary material, further inquiries can be directed to the corresponding author/s.

## Ethics Statement

The studies involving human participants were reviewed and approved by the Ethics Committee of Beijing Jiaotong University. Written informed consent to participate in this study was provided by the participants' legal guardian/next of kin.

## Author Contributions

ZC and LQ: conception and design of study. LN, ZC, and LH: acquisition of data. LQ: analysis and/or interpretation of data. LQ and LN: drafting the manuscript. ZC and LH: revising the manuscript critically for important intellectual content. ZC, LQ, LN, and LH: approval of the version of the manuscript to be published. All authors contributed to the article and approved the submitted version.

## Funding

This article was a phased achievement of the science popularization project of public health under the background of COVID-19 (2020JBWZ001) funded by the basic research expenses of Universities affiliated to the central government.

## Conflict of Interest

The authors declare that the research was conducted in the absence of any commercial or financial relationships that could be construed as a potential conflict of interest.

## Publisher's Note

All claims expressed in this article are solely those of the authors and do not necessarily represent those of their affiliated organizations, or those of the publisher, the editors and the reviewers. Any product that may be evaluated in this article, or claim that may be made by its manufacturer, is not guaranteed or endorsed by the publisher.
